# Purification, Structural Analysis and Bioactivity of *Pueraria montana* Polysaccharide

**DOI:** 10.3390/foods14081359

**Published:** 2025-04-15

**Authors:** Dandan Chen, Hongliang Yao, Xiang Qiu, Lang Xu, Yanghui Ou, Jianghui Xin, Shengjia Lu, Mengjie Li, Yan Geng, Yali Zhang, Minxiu Hu, Zhiming Ren, Jia-Qiang Wu

**Affiliations:** 1 School of Pharmacy and Food Engineering, Wuyi University, Jiangmen 529020, China; chen15188581741@163.com; 2 Guangdong Key Laboratory of Animal Conservation and Resource Utilization, Institute of Zoology, Guangdong Academy of Sciences, Guangzhou 510260, China; yaohl@giz.gd.cn (H.Y.); qxiang5202023@163.com (X.Q.); xulang20220720@163.com (L.X.); ouyh0807@gmail.com (Y.O.); 18672222553@163.com (J.X.); 15123186435@163.com (S.L.); lmj100800@163.com (M.L.); 18797822920@163.com (Y.G.); zhangyl@giz.gd.cn (Y.Z.); resourcehmx@163.com (M.H.); 3 JiangMen Industrial Technology Research Institute, Guangdong Academy of Sciences, Jiangmen 529020, China

**Keywords:** *Pueraria montana*, polysaccharide, structure, antioxidant, anti-inflammatory, anti-aging

## Abstract

*Pueraria montana* is a medicinal and edible plant widely distributed in Asia. It has antipyretic, analgesic, and anti-inflammatory properties. In this study, a novel polysaccharide (PMPS-A_1_) was obtained through purification, and its biological activity was investigated. Structural analysis revealed that PMPS-A_1_ was composed of fructose and glucose, with a molecular weight of 12168 Da. The main chain structure was →1)-β-D-Fru*f*-(2→, →4)-α-D-Glc*p*-(1→, →4)-α-D-Glc*p*-(1→, and →4)-α-D-Glc*p*-(1→. The branched chain of α-D-Glc*p*-(1→3)-α-D-Glc*p*-(1→ and α-D-Glc*p*-(1→ connected to the *O*-3 and *O*-6 positions of residue →3,4)-α-D-Glc*p*-(1→ and →4,6)-α-D-Glcp-(1→, respectively. In vitro, PMPS-A_1_ had a favorable scavenging ability of the hydroxyl radical and 1,1-diphenyl-2-picrylhydrazyl (DPPH) radical and downregulated the expression of interleukin-6 and nitric oxide in lipopolysaccharide-induced RAW264.7 macrophages. In addition, the Caenorhabditis model assay demonstrated that PMPS-A_1_ decreased the buildup of lipofuscin and reactive oxygen species. Overall, these results enhance our knowledge of the chemical composition and bioactivity of a *Pueraria montana* polysaccharide and point to the potential use of PMPS-A_1_ for antioxidant and anti-aging qualities, providing a theoretical basis for the medicinal and edible application of *Pueraria montana* polysaccharide.

## 1. Introduction

*Pueraria montana* belongs to the Leguminosae and is widely distributed in China, Laos, Thailand, Myanmar, Bhutan, and other Asian countries. For thousands of years, people in East and Southeast Asia have used its roots (called “fenge”) as food and herbal medicine [1]. Two types of Puerariae, *Puerariae lobatae radix* and *Pueraria montana,* are listed in the *Chinese Pharmacopoeia* [2]. Studies have shown that *Puerariae lobatae radix* has anti-diabetes [3], anti-hypertension [4], anti-oxidation [5], anti-osteoporosis, and immunomodulatory activities [6]. It also contains many bioactive substances, such as isoflavones, polysaccharides, amino acids, and terpenes. Because of its strong medical efficacy and nutritional qualities, it has been referred to as “Asian ginseng” or “longevity powder” [7]. In the food industry, *Pueraria montana* can be used for starch production and subsequent processing into noodle products. Early ethnopharmacology documentation revealed that *Pueraria montana* had the potential to alleviate symptoms such as fever, diarrhea, and vomiting [8]. *Pueraria montana* has also been proven to alleviate cardiovascular disease, exhibit anti-diabetes effects, and protect the liver [9]. Polysaccharides are one of the important active ingredients in *Pueraria montana.*

Polysaccharides have a variety of health-promoting effects, including antioxidant, anti-inflammatory, immunomodulatory, anti-fatigue, anti-hypertensive, and hypoglycemic activities [10]. As people age, the production of oxygen free radicals in the body increases, while the ability to remove these free radicals gradually decreases. This leads to the progressive accumulation of reactive oxygen species (ROS) [11], which causes oxidative damage to the organism, Polysaccharides (such as lentinan, ganoderma lucidum polysaccharides, seaweed polysaccharides, etc.) contain active hydroxyl groups (−OH) or other functional groups that can directly bind to free radicals (such as −OH, O_2_−, etc.), neutralizing their oxidative activity. For example, β-glucan can scavenge free radicals through a hydrogen atom transfer mechanism. [12]. Cytokines like interleukin-6 (IL-6) [13], interleukin-1β (IL-1β), and tumor necrosis factor-α (TNF-α) [14] are key molecules involved in various physiological and pathological processes, especially inflammatory responses. Polysaccharides (such as lentinan and astragalus polysaccharides) can promote the transformation of macrophages from the pro-inflammatory M1 phenotype to the anti-inflammatory M2 phenotype, reduce the release of pro-inflammatory cytokines (TNF-α, IL-6, IL-1β), and increase the secretion of anti-inflammatory cytokines (IL-10, TGF-β) [15,16]. In previous research, people mostly studied the *Puerariae lobatae radix* polysaccharide but seldom focused on *Pueraria montana* polysaccharide.

Therefore, in this study, the polysaccharide of *Pueraria montana* (PMPS) was prepared by hot water extraction and ethanol precipitation, and the extraction process of PMPS was optimized through a single-factor experiment and the response surface method. Then, homogeneous polysaccharide PMPS-A_1_ was purified from diethyl aminoethyl cellulose 52 (DEAE-52) and Sephadex G-150 column chromatography. PMPS-A_1_ was characterized by high-performance gel permeation chromatography (HPGPC), high-performance ion chromatography (HPIC), ultraviolet–visible (UV-vis) spectroscopy, Fourier-transform infrared (FT-IR) spectroscopy, gas chromatography–mass spectrometry (GC-MS), and nuclear magnetic resonance (NMR) spectroscopy. Finally, the antioxidant, antibacterial, and anti-inflammatory activities of PMPS-A_1_ were investigated in vitro. The impact of PMPS-A_1_ on aging was investigated in vivo using the Caenorhabditis model.

## 2. Materials and Methods

### 2.1. Materials and Reagents

*Pueraria montana* was produced in Guangdong. Sowing was carried out in spring. The seeds were soaked in warm water for 24 h before sowing to promote germination. The row spacing was 1–1.5 m, and the plant spacing was 0.5–1 m. The seeds were sown at a depth of 2–3 cm. Subsequently, they were watered and fertilized, the weeds were removed, and supports were set up. The sample was dried at 60 °C, placed in a mortar, ground with a pestle into powder, and sieved through a 200-mesh sieve, and the obtained sample was sealed and stored in a cool place The total antioxidant capacity detection kit; TNF-α, IL-1β, and IL-6 ELISA kits; and malondialdehyde (MDA) were purchased from Shanghai Beyotime Biotechnology Co., Ltd. (Shanghai, China).

### 2.2. Extraction and Determination of Polysaccharides

Fresh PMPS was dried at 60 °C, ground into powder, and boiled at 80 °C in deionized water for 4 h, with a liquid/solid ratio of 10–50 (g/mL) (1 g powder). This procedure was repeated three times in total, and the supernatant was obtained by centrifugation at 4000 rpm for 10 min. Then, the supernatant was collected and concentrated at 80 °C to one-third (10–50 mL) of the volume of the collection solution. The solution was concentrated and precipitated four times with anhydrous ethanol at 4 °C for 24 h. The precipitates were separated by centrifugation (4000 rpm, 20 min) and then dissolved back into deionized water to choose the appropriate volume. PMPS was purified by column chromatography. The phenol–sulfuric acid method (using glucose as the standard) [17], Bradford method of measurement (using bovine serum albumin as the standard) [10], and sulfuric acid–carbazole method (using galacturonic acid as the standard) [18] were used to identify the contents of total sugar, protein, and uronic acid, respectively. The extraction yield of PMPS (Y_1_, %) is expressed as Y_1_ = M_1_/M_2_ × 100, where M_1_ is the mass of lyophilized PMPS (pre-freezing at −80 °C for 4 h, followed by a sublimation drying stage from −45 to about 30 °C for about 30 h, Haier Biomedical, Qingdao, China), and M_2_ is the quality of the raw powder.

### 2.3. Optimization of Polysaccharide Extraction

A single-factor design was adopted for exploring preliminary range for the extraction variables [19]. The effects of liquid-to-material ratio (A1: 10, 20, 30, 40 and 50 mL/g), extraction time (A2: 1 h, 2 h, 3 h, 4 h and 5 h), and extraction temperature (A3: 60 °C, 70 °C, 80 °C, 90 °C and 100 °C) on the yield of polysaccharides were examined.

### 2.4. Experimental Design of the Response Surface Methodology (RSM)

A three-variable, three-level 17-run Box–Behnken Design (BBD) was built using Design Expert software to ascertain the ideal ranges of the extraction variables (Table 1 and Table 2) based on the findings of the single-factor trials. This quadratic polynomial model was used to fit the experimental data [19]. The second-order polynomial equation is as follows:

(1) $$Y_{1}=X_{0}+\sum_{i=1}^{3}X_{i}A_{i}+\sum_{i=1}^{3}X_{ii}A_{i}^{2}+\sum_{i=1}^{3}\sum_{j=1}^{3}X_{i,j}A_{i}A_{j}$$

### 2.5. Purification of Crude Polysaccharides

The temperature for the hot water extraction method was set at 90 °C, the extraction time was 2.96 h, and the liquid–solid ratio was set at 29.86 (mL/g). Purification was performed with this extraction method. Sevage reagent was used to separate free proteins. This reagent and the polysaccharide solution had a 1:4 ratio. The mixture was kept at 30 °C in a shaking water bath with a constant temperature for 30 to 60 min. To remove salts and other tiny molecules, the centrifuged supernatant (MWCO 3500 Da) was dialyzed in distilled water for 48 h. Additionally, polysaccharides from *Pueraria montana* (PMPS) were obtained by freeze-drying and subsequently sent to be purified using the DEAE-52 column (5.5 × 40 cm, Beijing Ruida Henghui Technology Development Co., Ltd., Beijing, China). A gradient solution of distilled water and NaCl (0.1, 0.2, 0.3, and 0.5 mol/L) was used for elution, with a flow rate of 4 mL/min. Every three minutes, samples were taken, and the phenol–sulfuric acid method was used to calculate the total amount of sugar. Tubes that had the same elution peak were gathered and dialyzed for 48 h (MWCO 3500 Da), followed by freeze-drying to produce PMPS-A. Next, 20 mL of deionized water was added to 220 mg of PMPS-A. Then, a Sephadex G-150 (1.0 × 50 cm, BoRui Saccharide Biotech Co. Ltd., Yangzhou, China) column was used to purify PMPS-A. A gradient solution of distilled water was used to perform the elution process. Tubes that had the same elution peak were gathered and dialyzed for 48 h (MWCO 3500 Da), followed by freeze-drying to produce PMPS-A_1_.

### 2.6. Structural Characterization of PMPS-A_1_

#### 2.6.1. Molecular Weight Analysis

According to Zhang et al.’s method, we made minor modifications, and the specific operation method is as follows: An accurately weighed amount of 5 mg of PMPS-A_1_ was dissolved in 1 mL of mobile phase (0.05 mol/L of NaCl solution) by vortexing and centrifuged (12,000 rpm, 10 min). After filtering through a 0.22 μm aqueous microporous membrane, PMPS-A_1_ was placed in a 1.8 mL injection vial. HPGPC was used to analyze the solution at a column temperature of 40 °C and a flow rate of 0.7 mL/min [20]. A BRT105-103-101 tandem gel column (8 × 300 mm) and a difference detector RID-20A (Shimadzu, Kyoto, Japan) were used in the HPGPC system.

#### 2.6.2. Monosaccharide Composition Analysis

According to Zhang et al.’s method, we made minor modifications, and the specific operation method is as follows: An amount of 5 mg of PMPS-A_1_ was placed in an ampoule. After 2 mL of 3M TFA was added, PMPS-A_1_ was hydrolyzed for three hours at 60 °C [21]. The acid hydrolysis solution was transferred to a tube after being accurately absorbed. It was then blow-dried using nitrogen and thoroughly mixed with 5 mL of water. After 950 µL of deionized water was added to 50 µL, the mixture was centrifuged for five minutes at 12,000 rpm. The supernatant was used for IC analysis. An electrochemical detector and a Dionex Carbopac TM PA20 column (3 × 150 mm, Thermal Scientific, Waltham, MA, USA) were utilized for the HPIC analysis.

#### 2.6.3. Methylation Analysis

According to Zhang et al.’s method, we made minor modifications, and the specific operation method is as follows: PMPS-A_1_ (2–3 mg) was weighed, and 1 mL of anhydrous DMSO was added. Methylation reagent A (anhydrous alkali) solution was quickly added and dissolved, followed by the addition of methylation reagent B (iodomethane) solution. The reaction was conducted at 30 °C for 60 min. Then, 2 mL of ultra-pure water was added to the mixture to terminate the methylation reaction. The mixture was freeze-dried after 24 h of dialysis with a 1000 Da dialysis bag. After dialysis, 2 and 200 mg of KBr samples were accurately weighed and mixed, pressed into tablets, and scanned and recorded in a Fourier-transform infrared spectrometer (FT-IR650). Subsequent experiments were conducted after confirming the completion of methylation. The methylated PMPS-A_1_ was taken, and 1 mL of 2 mol/L TFA was added for hydrolysis for 90 min. The mixture was evaporated to dryness using a rotary evaporator. Two milliliters of double-distilled water was added to the residue, which was then reduced with 60 mg of NaBH_4_ for 8 h, neutralized with glacial acetic acid, spin-evaporated, and dried in a 101 °C oven. Finally, 1 mL of acetic anhydride was added for acetylation at 100 °C for 1 h and then cooled. Then, 10 mL of pure water was added to terminate the reaction. The acetylated product was dissolved in 3 mL of CH_2_Cl_2_ and transferred to a separatory funnel. A small amount of distilled water was added and shaken thoroughly to remove the upper aqueous solution. This process was repeated 4 times. The CH_2_Cl_2_ layer was dried with an appropriate amount of anhydrous sodium sulfate, concentrated to 1 mL, and placed in a liquid-phase vial. The resulting partially methylated alditol acetates (PMAAs) were then determined using a gas chromatography–mass spectrometer (GC-MS; 13,000–7000, Thermo Scientific, Waltham, MA, USA) on an HP-INNOVAX chromatographic column (30 m × 0.32 mm × 0.25 um). The program heating conditions were as follows: a starting temperature of 140 °C and heating up to 230 °C at 1 °C/min. The inlet temperature was 250 °C, the detector temperature was 250 °C, the carrier gas was helium, and the flow rate was 1 mL/min.

#### 2.6.4. UV–Vis Spectroscopy Analysis

PMPS-A_1_ was scanned between 200 and 500 nm using an ultra-micro-ultraviolet spectrophotometer (NP80 Touch, Munich, German) at a concentration of 1 mg/mL.

#### 2.6.5. Congo Red Analysis

According to Wang et al.’s method, we made minor modifications, and the specific operation method is as follows: An 80 µmol/L Congo red solution was combined with PMPS-A_1_ (3 mg/mL), and then 1 mol/L NaOH solution was added. NaOH was finally found to be at concentrations of 0, 0.05, 0.1, 0.15, 0.2, 0.3, and 0.4 mol/L. Following five minutes of standing, an ultra-micro-ultraviolet spectrophotometer (NP80 Touch, Germany) was used to measure the solution’s maximum absorption wavelength, which was in the 400–600 nm range [22].

#### 2.6.6. FT-IR Spectroscopy Analysis

PMPS-A_1_ was mixed with KBr powder and thoroughly ground to form a compression plate, which was measured in the spectrum of 400–4000 cm^−1^ by TENSOR 27 FT-IR spectrometer (Bruker, Berlin, Germany).

#### 2.6.7. Scanning Electron Microscope (SEM) Analysis

According to Shen et al.’s method, we made minor modifications, and the specific operation method is as follows: PMPS-A_1_ was freeze-dried, quenched to expose its cross-section, and adhered with its surface and cross-section upwards onto a copper platform. Gold was sputtered on an ion sputtering instrument for 5 min, and the surface morphology of PMPS-A_1_ was detected by scanning electron microscopy (Sigma-300, Berlin, Germany) [23].

#### 2.6.8. Thermal Analysis

According to Zhang et al.’s method, we made minor modifications, and the specific operation method is as follows: A synchronous thermal analyzer (Mettler TGA/DSC3, Greifensee, Switzerland) produced by Mettler Toledo was used to study the thermal properties of PMPS-A_1_, based on thermogravimetric (TG) and differential thermogravimetric (DTG) methods. PMPS-A_1_ (10 mg) was placed on Al_2_O_3_ aluminum, using empty aluminum as the raw material, and experiments were conducted at a heating rate of 10 °C/min in an N_2_ environment at a temperature of 30–500 °C [24].

#### 2.6.9. NMR Spectroscopy Analysis

An amount of 50 mg of PMPS-A_1_ was weighed, dissolved in 0.5 mL of D_2_O (99.9%, Luoen, RH53622), and then freeze-dried. The freeze-dried powder was then dissolved in 0.5 mL of heavy water and freeze-dried again, with this process repeated to fully exchange active hydrogen. The sample was dissolved in 0.5 mL of heavy water and placed at a room temperature of 25 °C to measure the ^1^H NMR spectrum, ^13^C NMR spectrum, DEPT-135 one-dimensional spectrum (which provides important support for molecular structure analysis by distinguishing carbon types, simplifying spectra, and enhancing sensitivity), and two-dimensional spectrum (COSY, HSQC, HMBC, NOESY), using a 600 MHz NMR (Bruker, Germany) nuclear magnetic resonance instrument, C_3_D_6_O (Cambridge Isotope Laboratories, PR-32722) as an internal reference. The chemical shift value used for calibrating the obtained spectrum is 30.89 ppm 13C is 150.91 MHz, with a sampling frequency of 1024 times and a relaxation time of 2 s; 1H has a resonant frequency of 600 MHz and a sampling frequency of 16 times. The NMR results were processed using MestReNova software.

### 2.7. In Vitro Antioxidant Activity of PMPS-A_1_

#### 2.7.1. DPPH Radical Scavenging Assay

Vitamin C (Vc) was used as a positive control. Different concentrations of PMPS-A1, ranging from 1 to 5 mg/L, were set as sample groups. The experiment was divided into a measurement group (80 µL sample group + 120 µL reagent one), a control group (80 µL sample group + 120 µL extraction solution), and a blank group (80 µL extraction solution + 120 µL reagent one), which were added dropwise to a 96 well plate. Then, an enzyme-linked immunosorbent assay (ELISA) reader (Tecan M2001, Tecan, Mannedorf, Switzerland) was implemented to detect the absorbance at 515 nm after 30 min at room temperature and dark exposure. According to the method used for the kit provided by Shanghai Enzyme-linked Biotechnology Co., Ltd., Shanghai, China, the calculation formula was as follows:

DPPH radical scavenging rate (%) = (A_b_ − A_m_/A_b_) × 100% (2)

where A_b_ represents the absorbance value of the blank control in the working fluid, and A_m_ represents the absorbance value of the sample in the working fluid.

#### 2.7.2. Hydroxyl Radical Scavenging Assay

According to the usage method of the reagent kit provided by Shanghai Enzyme linked Biotechnology Co., Ltd., the working solution was diluted in a ratio of 1:9 (mother liquor: distilled water) to prepare reagent four. Reagent one, reagent two, and reagent three were prepared in a ratio of 2:1:2. The sample was set to a gradient concentration of 1–5 mg/mL PMPS-A1. A measurement group was set up (50 µL working solution + 50 µL sample + 50 µL reagent 4), a control group was set up (50 µL working solution + 50 µL distilled water + 50 µL reagent 4), and a blank group was set up (50 µL working solution + 100 µL distilled water). These were mixed, incubated at 37 °C for 60 min, and then centrifuged at 25 °C for 5 min with a centrifugal force of 8000 g, and 200 µL was aspirated into a 96 well plate at 536 nm using a microplate reader Tecan M2001, Tecan, Mannedorf, Switzerland) to measure absorbance. The calculation formula was as follows:

Hydroxyl radical scavenging rate (%) = [(A_m_ − A_c_)/A_b_ − A_c_] × 100% (3)

where A_m_, A_c_, and A_b_ were the absorbance values of the measurement group, control group, and blank group, respectively.

### 2.8. In Vitro Anti-Inflammatory Activity of PMPS-A_1_

#### 2.8.1. Cell Viability Assay

RAW 264.7 cells were seeded in 96-well plates and treated with PMPS-A_1_ at different concentrations (1.25, 2.5, 5 mg/mL) for 24 h. Fresh culture medium containing 10% of CCK-8 was added to each well. The plates were then incubated at 37 °C for 1 h. By using a microplate reader (Tecan M2001, Thermo Fisher, Waltham, MA, USA), the absorbance was measured at 450 nm. The formula that follows was used to determine cell viability:

Cell viability = [(A_s_ − A_b_)/(A_c_ − A_b_)] × 100% (4)

where A_s_ indicates the sample’s absorbance, A_b_ represents the blank’s absorbance, and A_c_ indicates the control’s absorbance.

#### 2.8.2. Determination of Cytokines

RAW264.7 cells were seeded into 12-well plates at a density of 3 × 10^5^ cells per well and cultured at 37 °C in a humidified atmosphere with 5% CO_2_ overnight. Experimental groups included the blank control group (no lipopolysaccharide (LPS) stimulation), the positive control group (dexamethasone (DEX), 1 micromole), the LPS stimulation group (1 ug/mL), and treatment groups (PMPS-A_1_). The cells were pre-treated with compounds for 2 h. All experimental groups, except the blank control group, were induced with 1 μg/mL LPS for 4 h and LPS from *Escherichia coli* O55:B5 purified by phenol extraction.

### 2.9. Antibacterial Activities of PMPS-A_1_ Polysaccharide Extracted from Pueraria Montana

The agar well diffusion method was used to measure the antibacterial activity of PMPS-A_1_ at four distinct concentrations (20, 25, 30, and 35 mg/mL). *E. coli* 25922and Methicillin-resistant Staphylococcus aureus (MRSA) ATCC 29213, with approximately 1.5 × 10^8^ cfu/mL of columnar bacteria, were inoculated onto the surface of agar plates. Agar medium was perforated with 7 mm diameter holes. Different concentrations of PMPS-A_1_ solution were added to each well. Penicillin (100 ug/mL) was used as a positive control to determine the results. The plate was allowed to stand for 4 h at 4 °C to allow for the diffusion of PMPS-A_1_ in the agar, followed by incubation at 37 °C for 24 h. The antibacterial activity was evaluated by measuring the inhibition zone diameters (including a well diameter of 7 mm). All tests were carried out in triplicate, and the values presented are the average of three replicates [25].

### 2.10. Evaluating the Impact of PMPS-A_1_ on Aging in C. elegans

#### 2.10.1. Cultivation of *C. elegans*

Growing on nematode growth medium (NGM) plates, all C. elegans were infected with *E. coli* OP50 at 20 °C. At 37 °C, the *E. coli* was grown for 12 h. A bleaching solution was employed to obtain the eggs, and they were then washed with M9 buffer. Following a 48 h synchronization period, L4 nematodes were ready for the ensuing tests.

#### 2.10.2. Determining the Levels of Reactive Oxygen Species (ROS)

The synchronized L4 nematodes were split into four groups: the blank group and the PMPS-A_1_ administration group (1 mg/mL, 3 mg/mL, and 5 mg/mL). Then, 48 h following delivery, the nematodes were incubated for 15 min at a concentration of 10 mmol/mL H_2_O_2_. Every worm was collected and given three rounds of washing with M9 buffer. Worms were subjected to a 10 mM DCFH-DA fluorescent probe dye solution (Biyuntian, Shanghai, China) for 30 min at 37 °C in order to measure the ROS level. The relative intensity of ROS fluorescence was measured and examined using the fluorescent microscope (EVOS, Thermometer Fisher Scientific, Waltham, MA, USA) and ImageJ software 1.54 (NIH, Bethesda, Rockville, MD, USA).

#### 2.10.3. Lipofuscin Accumulation Assay

L4 synchronized nematodes were chosen to be placed on an NGM plate with PMPS-A_1_ and OP50. Following two days of treatment, the nematodes were moved to a centrifuge tube filled with M9 buffer after being treated for fifteen minutes with 10 mmol/mL of H_2_O_2_. To precipitate nematodes, the centrifuge tube that was filled with them was centrifuged for two minutes at 3000 rpm. The nematodes were put on a 2% agarose plate after the supernatant was discarded. A fluorescent microscope was used to study them. After observing its shape in both light and dark fields, images were taken, and the fluorescence was measured using ImageJ. Graphpad Prism 8 was used to statistically assess the light intensity.

## 3. Results and Discussion

### 3.1. Single-Factor Experiments

**Figure 1:**
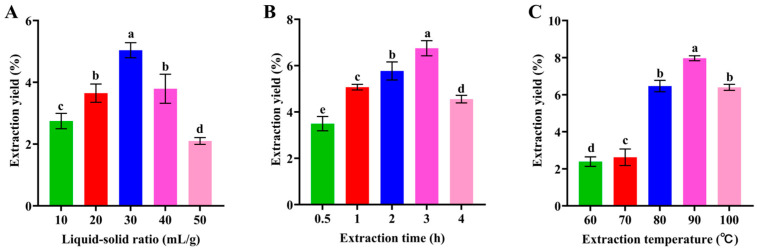
Effect of solid–liquid ratio (**A**), extraction time (**B**), and extraction temperature (**C**) on the yield of PMPS. The above values are expressed as mean ± SD (*n* = 3). Different letters indicate (a, b, c, d and e) significance, *p* < 0.05. PMPS, *Pueraria montana* polysaccharides.

As shown in Figure 1A, the yield of polysaccharides significantly increased (*p* < 0.05) when the liquid–solid ratio increased. The concentration difference was more noticeable, and the diffusion of polysaccharide molecules happened quicker with a larger liquid-to-raw-material ratio. On the other hand, the polysaccharide extract rate peaked at 30:1 and subsequently began to decline [26]. In the presence of excess solvent, non-sugar substances were dissolved, and the dissolution of polysaccharides was hindered, leading to a decrease in total polysaccharide yield [27].

As shown in Figure 1B at 0.5–3 h, the yield of PMPS significantly increased and reached a maximum value at 3 h. In addition, longer extraction times could also cause structural degradation and disintegration [28].

### 3.2. Optimization of the Polysaccharides Extraction Conditions

#### 3.2.1. Statistical Analysis and Model Fitting

To optimize the extraction parameters and analyze the interactive effects of the three factors on the extraction rate, a BBD under RSM was used, as shown in Table 1 [19], with the highest Y1 of 9.77% measured at 30 (mL/g) for the liquid–solid ratio, 3 h for the extraction time, and 90 °C for the extraction temperature [29]. Multivariate regression analysis was used to derive an equation of second-order polynomial from the experimental information.

Y_1_ = 9.55 − 0.08A_1_ − 0.12A_2_ + 0.093A_3_ + 0.047A_1_A_2_ + 0.042A_1_A_3_ + 0.31A_2_A_3_ − 2.99A_1_^2^ − 2.00A_2_^2^ − 2.10A_3_^2^ (5)

Table 1 shows the ANOVA findings for this model. The fitted model’s R2 and Radj2 values were 0.9891 and 0.9952, respectively. The model was considered significant (*p* < 0.05) since its *p*-value was less than 0.001, and its *p*-value for the lack of fit was 0.73 [30]. The coefficients of the other parameters were not significant, but the interaction term coefficients (A_2_A_3_), monomial coefficients (A_2_), and quadratic term coefficients (A_1_^2^, A_2_^2^, and A_3_^2^) were. The polynomial model equation’s low coefficient of variation (3.82) demonstrated its high accuracy and dependability. As a result, the relationship between extraction temperature (A_3_) and extraction time (A_2_) had a substantial impact on PMPS of extraction and warranted careful study [29].

#### 3.2.2. Response Surface Analysis

**Figure 2:**
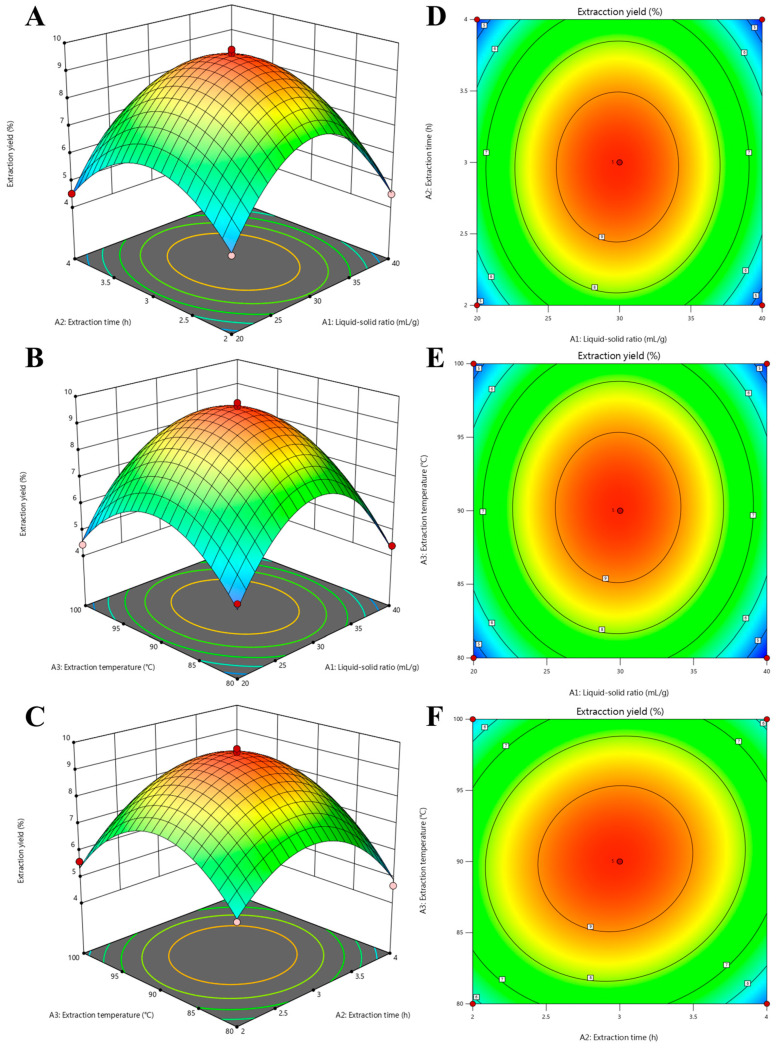
Response surface (**A**–**C**) and contour plots (**D**–**F**) showing the effect of liquid-to-material ratio (A_1_, mL/g), extraction time (A_2_, h), and extraction temperature (A_3_, °C) on the extraction yield of PMPS (Y_1_, %).

The produced 2D contour plots and 3D response surface show how the process variables relate to one another. The Y1 of PMPS (Figure 2) first increased when two variables (A2 and A3) increased, then decreased. At an extraction temperature of 90.20 °C and an extraction period of 2.97 h, the highest Y1 of PMPS was obtained. According to Table 2 and Figure 2, the interaction between A2A3 was stronger than the interactions between A1A2 and A1A3. Additionally, the denser the elliptical contour plot, the more substantial the interaction impact between the variables [31].

#### 3.2.3. Optimization and Verification

The numerical optimization findings show that the PMPS Y1 conditions were 90 °C for the extraction temperature, as shown by the ANOVA (Table 2) and the reaction surface diagrams (Figure 2A–C), 2.96 h for the extraction time, and 29.86 (mL/g) for the liquid–solid ratio. Under these circumstances, the model’s estimated Y1 for PMPS was 9.55%. The extraction temperature was set at 90 °C, the extraction time was set at 2.96 h, and the liquid–solid ratio was set at 29.86 (mL/g) in consideration of the experiments’ viability. To show the response surface technique model’s dependability, three replicate tests were carried out in real-world settings. With n = 3, the experimental Y1 of PMPS was 9.78 ± 0.09%. This shows that the BBD model is valid because it is near the expected value of 9.55% [32]. It was discovered that the optimized polysaccharide extraction yield was less than that of a partial BBD trial. Yet, compared to the single-factor experiment, the optimized extraction yield of PMPS was greater, demonstrating the excellent accuracy and efficacy of model [29].

### 3.3. Purification of PMPS-A_1_

**Figure 3:**
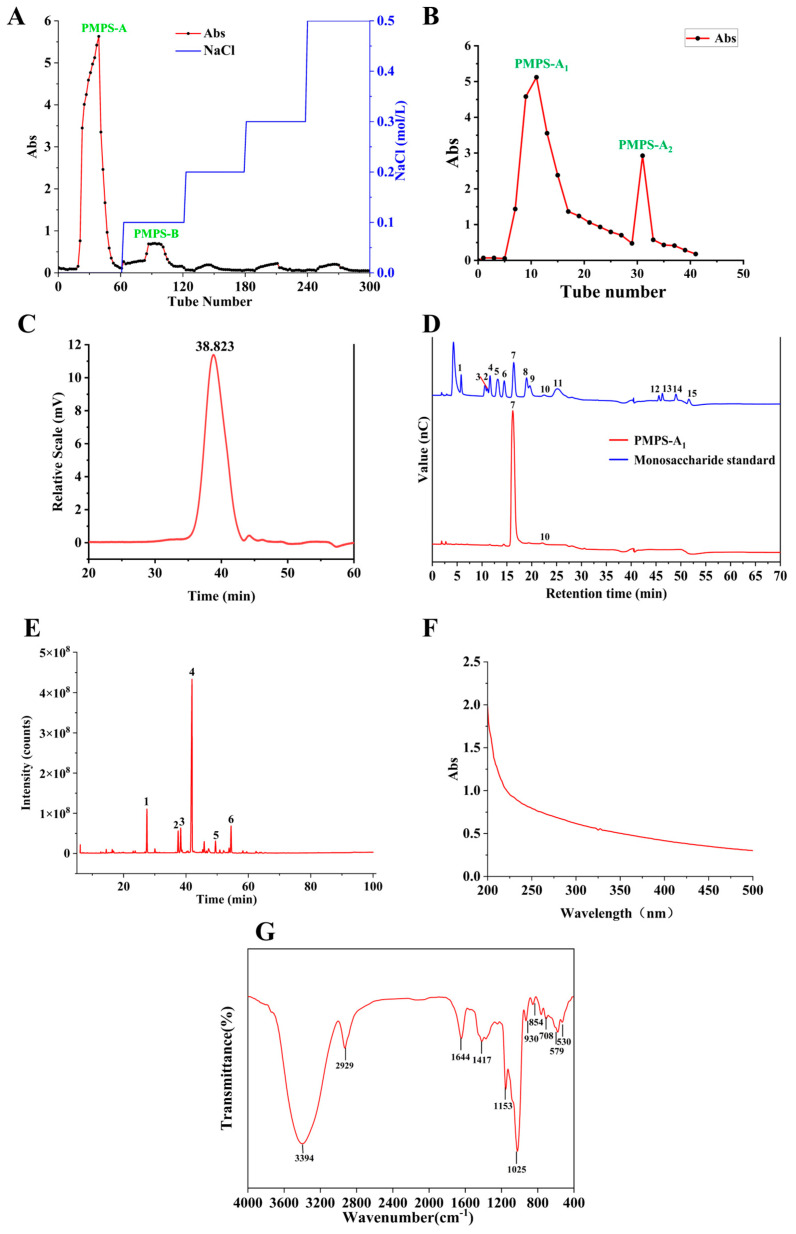
DEAE-52 elution curves (**A**); Sephadex G-150 elution curves analysis (**B**); HPGPC spectrum and peak Mw of PMPS-A_1_ (**C**); HPIC chromatograms of standard monosaccharides and PMPS-A_1_; 1, Fuc; 2, GalN; 3, Rha; 4, Ara; 5, GlcN; 6, Gal; 7, Glc; 8, Xyl; 9, Man; 10, Fru; 11, Rib; 12, GalA; 13, GulA; 14, GlcA; 15, ManA (**D**); Total ion chromatogram of PMPS-A_1_ (**E**); UV spectra of PMPS-A_1_ (**F**); FT-IR of PMPS-A_1_ (**G**).

As can be seen from Figure 3A–C, PMPS was purified using DEAE-52 cellulose and two fractions, named PMPS-A and PMPS-B. We identified PMPS-A, the polysaccharide with the highest absorbance, based on the ion column elution curve. About 95% of PMPS-A was made up of sugar. The Sephadex G-150 column was used to further purify PMPS-A [33]. It was employed to offer further purification. The peak with the highest polysaccharide content was determined based on its elution curve and named PMPS-A_1_. The HPGPC spectrum showed that the yield of PMPS-A_1_ purified by DEAE-52 cellulose and the Sephadex G-150 column was 53%, which was a single peak, indicating that the purity of PMPS-A_1_ was >99%. Using the phenol sulfuric acid method, the total sugar content inPMPS-A_1_ was about 99.08 ± 0.48%. PMPS-A_1_ contained 0.032 ± 0.0016% protein, and its uronic acid content was 0.6 ± 0.021%. This also indicates that PMPS-A_1_ is a neutral sugar.

### 3.4. Physicochemical Properties of PMPS-A_1_

#### 3.4.1. Analysis of PMPS-A_1_ Molecular Weight

The molecular weight and purity, polydispersity index, and mean molecular mass of PMPS-A_1_ were ascertained using the HPGPC method; its Mw was 12,198 Da, calculated using y = −0.1948x + 11.649R^2^ (R^2^ = 0.9947), its Mn was 12,168 Da (Figure 3C), and its polydispersity index was 1.00, indicating high uniformity of PMPS-A_1_ [29]. Our study is different from that of a polysaccharide (a-PLP, molecular weight of 22.675 kDa) isolated and purified from Pueraria lobata by Cai et al. [34].

#### 3.4.2. Analysis of PMPS-A_1_ Monosaccharide Composition

Using HPIC to determine the monosaccharide composition of PMPS-A_1_ (Figure 3D), the results indicate that PMPS-A_1_ was composed of glucose and fructose (glucose: fructose, 7:5). This is different from the polysaccharide purified by Du et al. [35] from *Puerariae thomsonii*, which consists entirely of glucose. This may be caused by different varieties

#### 3.4.3. Methylation Analysis of PMPS-A_1_

The methylation results of PMPS-A_1_ are shown in Table 3. PMPS-A_1_ had six glycosidic bonds, namely Glc*p*-(1 →, →1)-Fru*f*-(2→, →3)-Glc*p*-(1→, →4)-Glc*p*-(1 →, →3,4)-Glc*p*-(1→, and →4,6)-Glc*p*-(1→. Figure 3E shows the total ion chromatogram of PMPS-A_1_.

#### 3.4.4. Analysis of PMPS-A_1_ UV-Vis and FT-IR

Figure 3F shows that there is no clear absorption peak in PMPS-A_1_ at 260–280 nm, indicating that it does not have nucleic acid or protein. In the FT-IR spectrum in Figure 3G, the stretching vibration of O-H is responsible for the strong and broad absorption band seen at approximately 3394 cm^−1^ within the 4000–500 cm^−1^ range, whereas the stretching vibration of C-H is accountable for the small band at 2929 cm^−1^ [27]. These two bunches are typical polysaccharide bands. It has been established that the IR peak of absorption at 1644 cm^−1^ is caused by the stretching vibration of C=O [36]. The pyranose ring’s stretching vibration causes a prominent band in FT-IR between 930 and 1153 cm^−1^. The type α glycosidic linkages are present, as indicated by the absorption at 849 cm^−1^. Additionally, the absorption at 1025 and 1153 cm^−1^ shows that the sugar is pyranose [30].

#### 3.4.5. Analysis of the Thermal Properties of PMPS-A_1_

**Figure 4:**
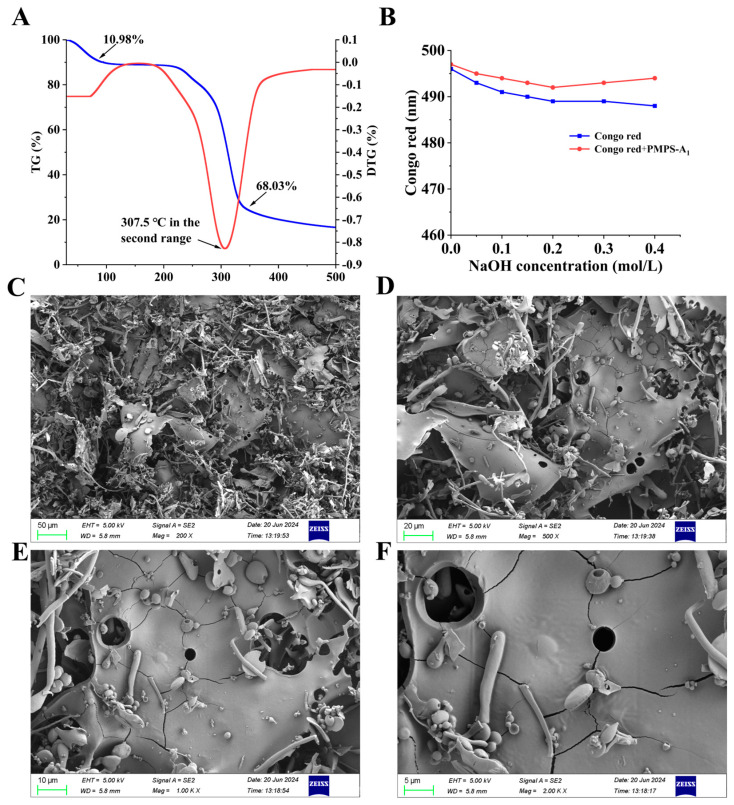
TG−DTG curves (**A**), blue line: TG; red line: DTG; Congo red experimental analysis of PMPSA_1_ (**B**); SEM (200×, 500×, 1.0 k×, 2.0 k×, magnification) (**C**–**F**).

In the food sector, thermal stability is a crucial physicochemical characteristic of polysaccharides [37]. TG and DTG are commonly used to evaluate the thermal properties of polysaccharides, and the figure shows the outcomes that were achieved. As Figure 4A illustrates, the decomposition process of PMPS-A_1_ is divided into three stages. The first stage is around 30–126.6 °C, with a 10.98% weight loss rate. A tiny amount of water will be adsorbed on PMPS-A_1_ at this point, as physical factors, because PMPS-A_1_ lose the water adsorbed on polysaccharides. The weight loss approaches 68.03% during the second stage. The maximum weight loss rate temperature is 307.5 °C. PMPS-A_1_ undergo intense decomposition within this temperature range, resulting in a significant decrease in the weight of PMPS-A_1_. In the third stage, the temperature range is 400–500 °C. During this stage, the weight change in PMPS-A_1_ tends to be gentle, which is a slow carbonization stage. Most samples decompose into ash and inorganic components during this stage. Therefore, PMPS-A_1_ could be used as a food additive, drug carrier, and stabilizer in food and industrial applications [38].

#### 3.4.6. Analysis Congo Red of PMPS-A_1_

Figure 4B shows the maximum absorption wavelength changes in PMPS-A_1_ and Congo red in solutions of NaOH (0–0.4 M) of varying strengths. Compared with Congo red, with the increase in NaOH concentration, the maximum absorption wavelength of PMPS-A_1_ did not display a noticeable red shift, indicating that PMPS-A_1_ does not have a triple helix structure [39].

#### 3.4.7. Analysis of PMPS-A_1_ SEM 

SEM is an effective technique for determining the morphological features of biopolymers, such as proteins, peptides, and polysaccharides [40]. In Figure 4C–F shows the SEM micrographs of PMPS-A_1_ at magnifications of 200×, 500×, 1.0 k×, and 2.0 k×. This esport image has a small number of holes, small spherical shapes, and stick-shaped shapes with scattered distribution.

#### 3.4.8. NMR Analysis of PMPS-A_1_

**Figure 5:**
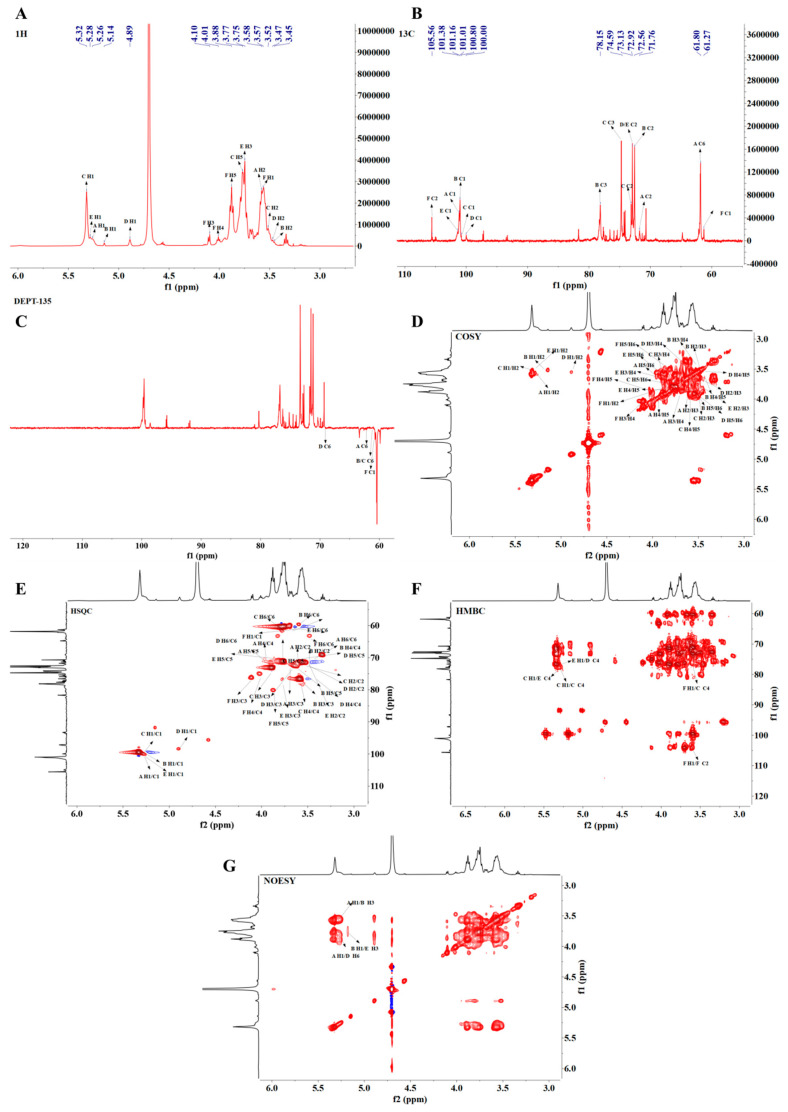
^1^H NMR (**A**), ^13^C NMR (**B**), DEPT −135 (**C**), COSY (**D**), HSQC (**E**), HMBC (**F**), and NOESY (**G**) spectra of PMPS-A_1_.

PMPS-A_1_’s 1D and 2D NMR spectra were displayed in Figure 5, and Table 4 lists the chemical shifts in PMPS-A1 at ^1^H (Figure 5A) and ^13^C (Figure 5B). The proton signals are primarily concentrated between δ 3.0 and 5.5 ppm. Signals at δ 3.2–4.0 ppm correspond to the sugar ring protons, while the main anomeric proton peaks at δ 4.87, 5.18, 5.25, 5.26, and 5.30 ppm are found in the δ 4.3–5.5 ppm range [41]. The carbon spectrum analysis in ^13^C NMR (201 MHz, D_2_O) shows that the carbon signals are primarily concentrated between δ 60 and 120 ppm. Observing the carbon spectrum reveals that the main anomeric carbon signal peaks at δ 99.48, 100.48, 100.86, 100.92, and 100.95 ppm, with δ 93 and 105 ppm being the primary range of the anomeric carbon area. Dept-135 spectrum analysis shows that the peaks at δ 61.27 64.81, 69.04, 61.48, 61.89, and 61.46 ppm are inverted peaks, indicating chemical shifts in glucose C6 or fructose C1.

To elucidate the hydrogen and carbon proton signals of each sugar residue in PMPS-A_1_, further analysis was conducted using 2D NMR spectroscopy. Six proton-related peaks of heteroatom carbon can be observed in the chemical shift region of the HSQC spectrum. Based on the methylation results, it can be determined that PMPS consists of six sugar residues labeled A, B, C, D, E, and F. According to the peak distribution of HSQC spectra, the chemical shifts in the heteroatom hydrogen and heteroatom carbon proton of residue A are δ 5.52 ppm and δ 100.92 ppm, respectively, indicating that residue A has an alpha configuration. The chemical shift in H2~H6 in residue A was determined by ^1^H-^1^H-COSY spectroscopy (Figure 2D), with cross peaks appearing at δ 5.25/3.59 (H1/H2), δ 3.59/3.69 (H2/H3), δ 3.69/3.94 (H3/H4), δ 3.94/3.96 (H4/H5), and δ 3.96/3.59 (H5/H6). Combined with HSQC spectra, the relative peaks of C1/H1, C2/H2, C3/H3, C4/H4, and C5/H5 indicate that the corresponding C1 to C5 are δ 100.92, 71.53, 73.62, 70.2, and 71.27 ppm, respectively. Based on these findings and literature data, residue A has been identified as α-D-Glc*p*-1→.

Residue B shows an end-group proton signal at δ 5.18. The peak at δ 5.18/100.86 in the HSQC spectrum is attributed to the H1/C1 of the end-group region, indicating that residue B has an α configuration. The 1H-1HCOSY spectrum shows cross-peaks at δ 5.18/3.48, 3.48/3.52, 3.52/3.42, 3.42/3.61, 3.61/3.66, and 3.66/3.73 ppm. The two correlated peaks between H5 and H6 indicate that the hydrogen atoms are in two different chemical environments, which is consistent with the H2-H6 signals of residue B at δ 5.18, 3.48, 3.52, 3.42, 3.61, and 3.66/3.77 ppm. Additionally, the cross-peaks observed at δ3.48/72.33, 3.52/78.02, 3.86/70.26, and 3.61/73.52 ppm correspond to H2/C2, H3/C3, H4/C4, and H5/C5, respectively. Based on these findings and literature data, residue A has been identified as →3)-α-D-Glc*p*-(1→. Using a similar analysis method, combined with HMBC and NOESY analysis, all glycosidic bond signals were identified, as detailed in Table 4.

In the HMBC spectrum, based on the one-dimensional and two-dimensional NMR spectra, we assigned the glycosidic bond signals of polysaccharides. The anomeric hydrogen of glycosidic bond →1)-β-D-Fru*f*-(2→ has a correlated signal peak with its own C2, indicating the existence of a linkage pattern of →1)-β-D-Fru*f*-(2→1)-β-D-Fru*f*-(2→. There is a correlation signal peak between the C2 of the glycosidic bond →1)-β-D-Fru*f*-(2 → and the H4 of →4)-α-D-Glc*p*-(1 →, indicating the existence of a linkage pattern of →1)-β-D-Fru*f*-(2 → 4)-α-D-Glc*p*-(1→. The anomeric hydrogen of glycosidic bond →4)-α-D-Glc*p*-(1→ has a related signal peak with its own C4; in addition, the anomeric carbon has a related signal peak with its own H4, indicating the existence of the linkage mode of → 4)-α-D-Glc*p*-(1 →4)-α-D-Glc*p*-(1→. There is a correlation peak between the anomeric hydrogen of →4)-α-D-Glcp-1→) and the C4 of the glycosidic bond of →3,4)-α-D-Glcp-(1→, indicating the existence of glycosidic bond →4)-α-D-Glc*p*-(1→3,4)-α-D-Glc*p*-(1→. The anomeric hydrogen of →3,4) -α-D-Glc*p*-(1→ and glycosidic bond →4,6)-α-D-Glc*p*-(1→ has a correlation peak at C4, indicating the presence of glycosidic bond →3,4)-α-D-Glc*p*-(1→4,6)-α-D-Glc*p*-(1 →.

Branch analysis: There is a correlation peak between the anomeric carbon of α-D-Glc*p*-(1→ and the H3 of glycosidic bond→3)-α-D-Glc*p*-(1→, indicating the presence of glycosidic bonds α-D-Glc*p*-(1→3)-α-D-Glc*p*-(1→. There is a correlation peak between the anomeric carbon of →3)-α-D-Glc*p*-(1→ and the H3 of glycosidic bond →3,4)-α-D-Glc*p*-(1→, Indicating the presence of glycosidic bonds →3)-α-D-Glc*p*-(1→3,4)-α-D-Glc*p*-(1→.

In the NOESY spectrum, the anomeric proton of →4)-α-D-Glcp-(1→shows a correlation peak with the H4 of →4,6)-α-D-Glc*p*-(1→, indicating the presence of the glycosidic linkage →4)-α-D-Glc*p*-(1→4,6)-α-D-Glc*p*-(1→. The anomeric proton of α-D-Glc*p*-(1→shows a correlation signal with the H6 of →4,6)-α-D-Glcp-(1→, demonstrating the linkage pattern of α-D-Glcp-(1→4,6)-α-D-Glcp-(1→.

**Figure 6:**
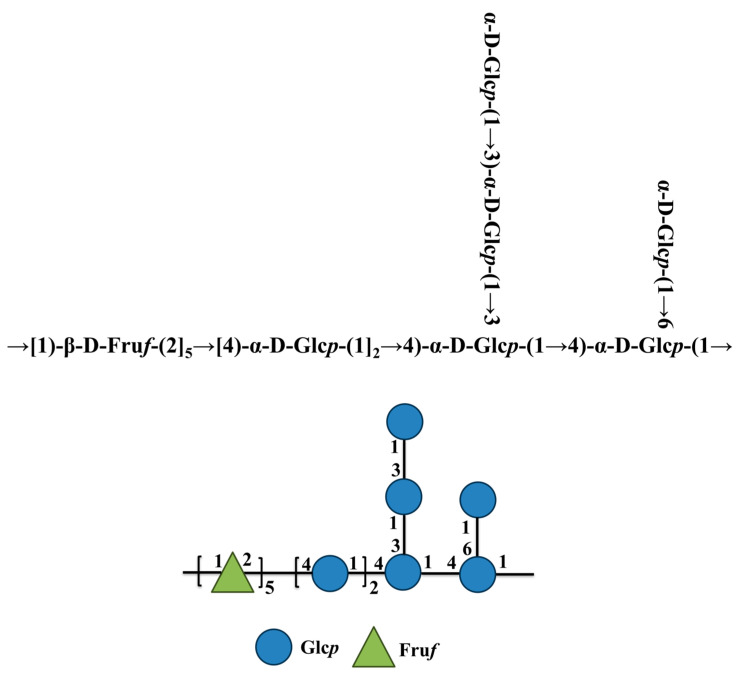
Schematic diagram of a possible structural model for PMPS-A_1_.

Based on the above description s and analysis of the monosaccharide composition ratio, as shown in Figure 6, it can be inferred that he main chain structure of PMPS-A_1_ is →1)-β-D-Fru*f*-(2→, →4)-α-D-Glc*p*-(1→, →4)-α-D-Glc*p*-(1→, and →4)-α-D-Glc*p*-(1→, and the branched chain of α-D-Glc*p*-(1→3)-α-D-Glc*p*-(1→, α-D-Glc*p*-(1→ connected to the main chain *O*-3 and *O*-6 position of residue →3,4)-α-D-Glc*p*-(1→ and →4,6)-α-D-Glc*p*-(1→.

### 3.5. In Vitro Analysis of the Antioxidant Activity of PMPS-A_1_

**Figure 7:**
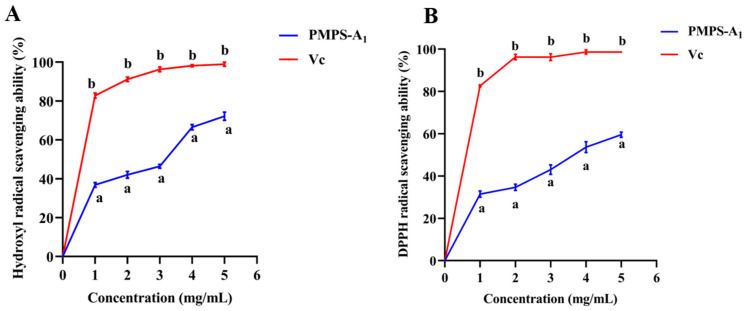
Scavenging rate of PMPS-A_1_ on hydroxyl radicals (**A**); scavenging effect of PMPS-A_1_ on DPPH free radicals (**B**). The above values are expressed as mean ± SD (n = 3). At the same concentration of PMPS-A_1_ compared with vitamin C, different letters (a, b) represent significance.

The molecular weight, monosaccharide composition, type, and conformation of the glucoside bond affect the activity of polysaccharides [42]. Antioxidant effects are one of the most important biological properties of pueraria polysaccharides, as they react with toxic free radicals, thereby preventing oxidative damage in the organism [43]. As shown in Figure 7A, PMPS-A_1_ scavenges hydroxyl radicals in vitro. As the concentration of polysaccharides rises, this effect gradually intensifies. When polysaccharide levels are 6 mg/mL, its ability to scavenge hydroxyl radicals is strong, with a clearance rate of 60%. At this concentration, Vc has a 100% scavenging activity against hydroxyl radicals [44].

The clearance rate is a measure of PMPS-A_1_’s capacity to scavenge DPPH free radicals in Figure 7B. The extract’s antioxidant activity increases with its clearance rate. The scavenging effectiveness of PMPS-A_1_ on DPPH free radicals is dose-dependent with polysaccharide concentration within a specific concentration range. The scavenging rate of polysaccharides on DPPH free radicals progressively rises with increasing polysaccharide concentration. Meanwhile, it can be observed that both PMPS-A_1_ and Vc have varying degrees of inhibitory effects on DPPH radicals, with Vc having a stronger inhibitory effect. Although not as effective as charged polysaccharides, neutral polysaccharides also have antioxidant properties [45]. It has been reported that water-soluble derivatives of bacterial glucan rennet composed of a →3)-β-D-Glcp-(1→ unit, laminaran containing →3)-β-D-Glcp-(1→ and →6)-β-D-Glc*p*-(1→ algal glucan, and →3)-β-D-Glc*p*-(1→ and →4)-β-D-Glc*p*-(1→, which are water-soluble derivatives of phytoglucan lichenan, had certain antioxidant activity [46]. It has also been reported that Radix Puerariae lobatae polysaccharide (PLP1) was composed of glucose, and the sugar residues are →4)-α-D-Glcp-(1→ and →4,6)-α-D-Glcp-(1→, had favorable scavenging ability of DPPH radical, superoxide anion radical, and ABTS radical [47]. In our study, PMPS-A_1_ was different from other *Puerariae lobatae radix* polysaccharides in that PMPS-A_1_ was composed of glucose and fructose, and the main chain was composed of →4)-α-D-Glc*p*-(1→, →3,4)-α-D-Glc*p*-(1→ and →4,6)-α-D-Glc*p*-(1→. It also included →1)-β-D-fru*f*-(2→, which may play a great role in the scavenging ability of DPPH free radicals and hydroxyl free radicals.

### 3.6. In Vitro Anti-Inflammatory Activity of PMPS-A_1_

In recent years, polysaccharides have attracted more and more attention from scientists all over the world because of their safe and effective anti-inflammatory and immunomodulatory effects [48]. Numerous physiological and pathological processes, including inflammation and the immunological response, are significantly mediated by cytokines [49]. When LPS activate macrophages, they release a variety of inflammatory mediators and factors, including TNF-α, NO, IL-6, and IL-1β [50].

**Figure 8:**
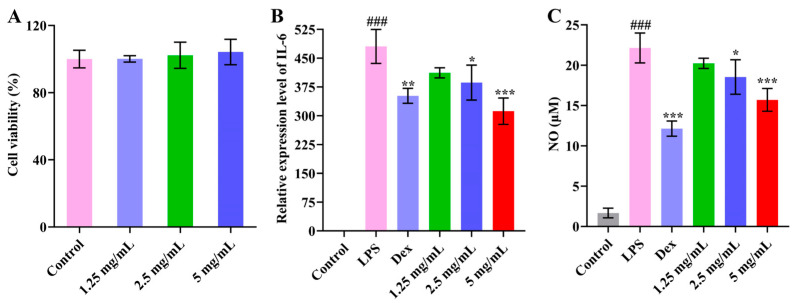
Effect of PMPS-A_1_ on cell viability of RAW264.7 macrophages (**A**). Effect of PMPS-A_1_ on the relative expression of IL-6 in RAW264.7 macrophages induced by LPS (**B**). Effect of PMPS-A_1_ on the relative expression of NO in RAW264.7 macrophages induced by LPS (**C**). Compared with control group, ^###^ *p* < 0.001; compared with LPS group, * *p* <0.05, ** p < 0.01, *** *p* < 0.001. The above values are expressed as mean ± SD (n = 3).

In order to examine PMPS-A_1_’s safety, we first carried out cytotoxicity tests. PMPS-A_1_ had no influence on cell viability when compared to the control group (*p* > 0.05) (Figure 8A). Both DEX and PMPS-A_1_ significantly (*p* < 0.05 or 0.001) downregulated IL-6 expression when compared to the LPS group (Figure 8B). Both DEX and PMPS-A_1_ considerably (*p* < 0.05 or 0.001) reduced the expression of NO dose-dependently in comparison to the LPS group (Figure 8C). It has been reported that low-molecular-weight polysaccharides (<30 kDa) have better water solubility and are easier to digest and absorb than high-molecular-weight polysaccharides (>100 kDa) [51]. Du et al. extracted and purified a water-soluble polysaccharide (GRP) from Glehniae radix with a molecular weight of 1.33 × 10^4^ Da [35]. The main chain of GRP consists of →6)-α-d-Glc*p*-(1→ and →3)-α-d-Glc*p*-(1→, and 1 out of every 14 residues of →6)-α-d-Glc*p*-(1→ and α-D-Glc*p*-(1→ branches are formed at the C-4 position. GRP downregulated the production of nitric oxide (NO) in a dose-dependent manner. In another study, the high-starch-content plant Pueraria mirifica of polysaccharide (PMP-2), with a molecular weight of 124.4 kDa, was mainly composed of arabinose and galactose [52]. PMP-2 was an Arabinogalactan, the main chain was composed of →6)-Gal*p*-(1→ and →4)-Gal*p*-(1→, and the side chain was composed of arabinose and rhamnose. PMP-2 pairs had certain immunomodulatory activities related to the release of NO, TNF-α, and IL-6. In our study, the molecular weight of PMPS-A_1_ was 12,168 Da; the monosaccharides were composed of glucose and fructose; the main chain was →1)-β-D-fru*f*-(2→, →4)-α-D-Glc*p*-(1→, →3,4)-α-D-Glc*p*-(1→, and →4,6)-α-D-Glc*p*-(1→; and the branch chain contained α-D-Glc*p*-(1→ and →3)-α-D-Glc*p*-(1→. The unique structure of PMPS-A1 against its anti-inflammatory activity may provide a solid foundation.

### 3.7. Antibacterial Activities of PMPS-A_1_

As shown in Table 5, the inhibition zone (10–14 mm) observed using different doses (from 20 to 35 mg/mL) gives exactly 50% of the inhibition zone for penicillin. The pharmacopeia’s classification aligns with clinical breakpoints, where an inhibition zone ≤50% of the reference antibiotic (penicillin) confirms resistance. Therefore, the tested PMPS-A_1_cannot be considered an effective antimicrobial agent against these strains under the current experimental conditions.

### 3.8. In Vivo Anti-Aging Experiment in C. elegans

#### 3.8.1. Analysis of the Experimental Results of PMPS-A_1_ on the Aging of *C. elegans*

**Figure 9:**
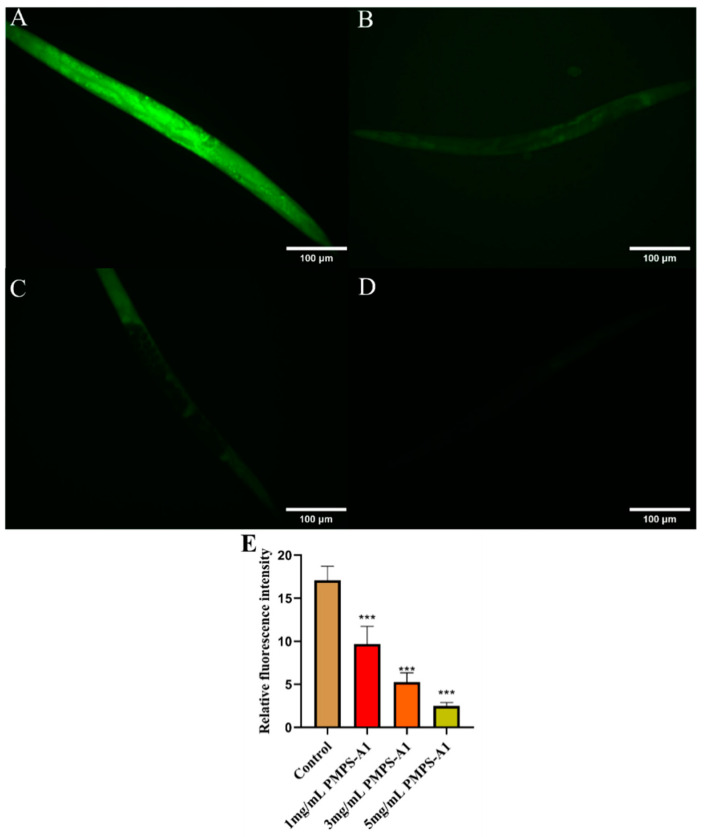
(**A**–**D**) are the ROS fluorescence levels of nematodes in the blank control group, 1mg/mL, 3mg/mL, and 5mg/mL, respectively. (**E**) Relative fluorescence intensity of ROS in nematodes. Compared with the blank group, *** *p* < 0.001. The above values are expressed as mean ± SD (n = 3).

ROS and organism aging are tightly connected. Appropriate reactive oxygen species can function as signaling molecules and take part in a range of physiological processes within cells, such as cell growth, differentiation, and apoptosis, while excessive reactive oxygen species can cause cell damage and senescence [53]. A popular fluorescent probe for measuring intracellular reactive oxygen species (ROS) levels is DCFH-DA (2′,7′-dichlorofluorescein diacetate) [54]. As shown in Figure 9. compared with the control group, the fluorescence accumulation level of ROS gradually decreased with the increase in PMPS-A_1_ concentration [55]. The evidence is clear that polysaccharides from *Pueraria montana* reduce ROS accumulation in nematodes, thereby delaying the extent of nematode senescence.

#### 3.8.2. PMPS-A_1_ Reduce Lipofuscin Accumulation in *C. elegans*

**Figure 10:**
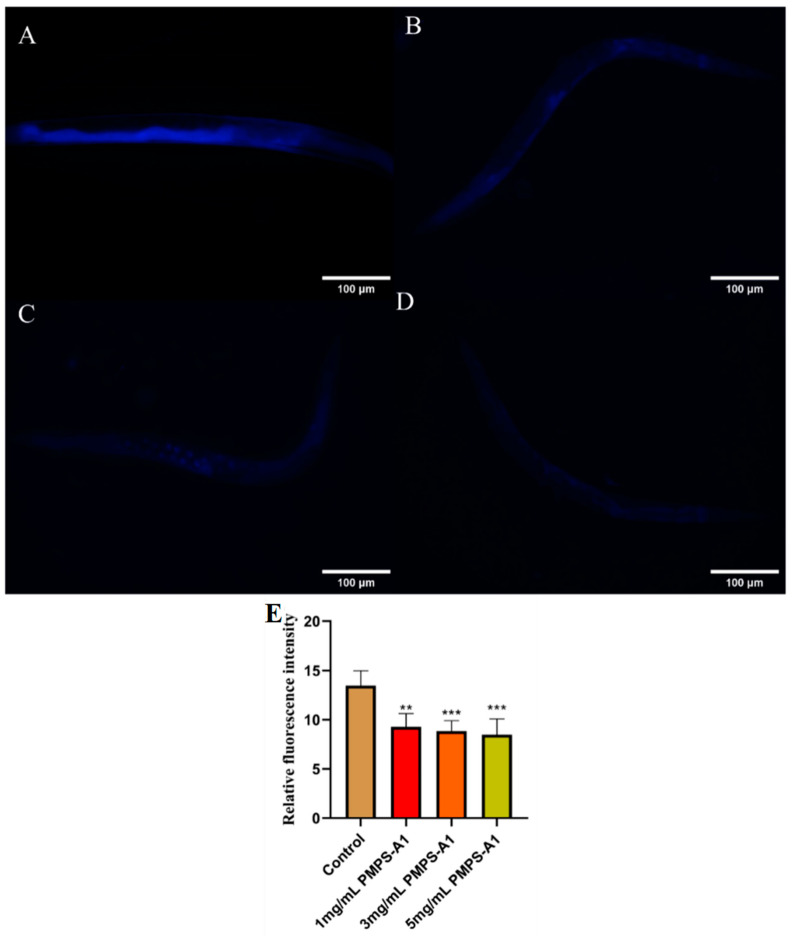
(**A**–**D**) Blue fluorescence produced by lipofuscin in nematodes of the blank control group, 1mg/mL, 3mg/mL, and 5mg/mL, respectively, under fluorescent irradiation. (**E**) Relative fluorescence intensity of lipofuscin in nematodes. ** *p* < 0.01,*** *p* < 0.001. The above values are expressed as mean ± SD (n = 3).

Lipofuscin often accumulates in neuron, cardiac, hepatic, and other tissue cells as people age, and it can also cause age spots to appear on the skin’s surface [56]. Reducing lipofuscin buildup may help postpone aging because it is strongly linked to the aging process. Nematodes’ spontaneous blue fluorescence of lipofuscin can be seen using a fluorescence microscope [57]. Compared with the blank control group, as shown in Figure 10, with the gradual increase in polysaccharide concentration, the fluorescence accumulation level of lipofuscin gradually decreases.

## 4. Conclusions

In this study, a unique neutral polysaccharide PMPS-A_1_ (Mw 12,168 Da) was obtained. The main chain structure is→1)-β-D-Fru*f*-(2→, →4)-α-D-Glc*p*-(1→, →4)-α-D-Glc*p*-(1→, and →4)-α-D-Glc*p*-(1→, and the branched chain of α-D-Glc*p*-(1→3)-α-D-Glc*p*-(1→, α-D-Glc*p*-(1→ connected to the main chain *O*-3 and *O*-6 position of residue →3,4)-α-D-Glc*p*-(1→ and →4,6)-α-D-Glcp-(1→, respectively. PMPS-A_1_ had favorable antioxidant and anti-inflammatory activities in vitro. The findings demonstrated that PMPS-A_1_ decreased the buildup of lipofuscin and reactive oxygen species. The above research provided a theoretical basis for the development and utilization of *Pueraria montana* polysaccharide. It also indicates that PMPS-A_1_ has untapped value in the food and beauty industry, for example, in functional foods used to develop foods that enhance immunity and anti-aging. Dietary supplements improve overall health. Its value to the beauty industry: it can protect the skin from environmental damage, promote skin repair and regeneration.

## Figures and Tables

**Table 1:** Box–Behnken experimental design and extraction of PMPS from *Pueraria montana*.

Entry	Coded Variable Levels			Yield (%)
	*A*_1_ ^a^	*A*_2_ ^a^	*A*_3_ ^a^	
1	−1 (1:20)	0 (3)	−1 (80)	4.68
2	−1 (1:20)	−1 (2)	0 (90)	4.73
3	−1 (1:20)	0 (3)	1 (100)	4.43
4	−1 (1:20)	1 (4)	0 (90)	4.52
5	1 (1:40)	−1 (2)	0 (90)	4.5
6	1 (1:40)	0 (3)	−1 (80)	4.39
7	1 (1:40)	0 (3)	1 (100)	4.31
8	1 (1:40)	1 (4)	0 (90)	4.48
9	0 (1:30)	1 (4)	−1 (80)	4.67
10	0 (1:30)	1 (4)	1 (100)	5.84
11	0 (1:30)	−1 (2)	−1 (80)	5.69
12	0 (1:30)	−1 (2)	1 (100)	5.6
13	0 (1:30)	0 (3)	0 (90)	9.51
14	0 (1:30)	0 (3)	0 (90)	9.77
15	0 (1:30)	0 (3)	0 (90)	9.47
16	0 (1:30)	0 (3)	0 (90)	9.35
17	0 (1:30)	0 (3)	0 (90)	9.64

^a^ *A*_1_, *A*_2_, and *A*_3_ denote the liquid-to-solid ratio (mL/g), extraction time (h), and extraction temperature (°C), respectively.

**Table 2:** Estimation of regression coefficient of quadratic polynomial model of PMPS yield and its significance analysis.

Source	Sum of Squares	DF	Mean Square	*F*-Value	*p*-Value
A_1_	0.0578	1	0.0578	1.03	0.3442
A_2_	0.1275	1	0.1275	2.27	0.1756
A_3_	0.0703	1	0.0703	1.25	0.3002
A_1_^2^	37.74	1	37.74	671.84	<0.0001 ***
A_2_^2^	16.78	1	16.78	298.74	<0.0001 ***
A_3_^2^	18.59	1	18.59	330.99	<0.0001 ***
A_1_A_2_	0.0090	1	0.0090	0.1606	0.7005
A_1_A_3_	0.0072	1	0.0072	0.1286	0.7305
A_2_A_3_	0.3969	1	0.3969	7.06	0.0326 *
Model	81.97	9	9.11	162.12	0.0001 ***
Residual	0.3933	7	0.0562		
Lack of fit	0.2888	3	0.0963	3.69	0.1200
Pure error	0.1045	4	0.0261		
Cor.total	82.36	16			
R^2^	0.9952				
R^2^_adj_	0.9891				
CV(%)	3.82				

Significance: * *p* < 0.05, *** *p* < 0.001.

**Table 3:** The methylation results of PMPS-A_1_.

Number	Type of Linkage	Methylated Sugars	Time (min)	Molar Ratio	Mass Fragments (*m*/*z*)
1	Glc*p*-(1→	2,3,4,6-Me_4_-Glc*p*	27.485	0.116	43, 71, 87, 101, 117, 129, 143, 161, 205
2	→1)-Fru*f*-(2→	3,5,6-Me_3_-Man*f*/Glc*f*	37.501	0.058	45, 71, 87, 99, 101, 129, 145, 161, 189
3	→3)-Glc*p*-(1→	2,4,6-Me_3_-Glc*p*	38.324	0.079	43, 87, 99, 101, 117, 129, 161, 173, 233
4	→4)-Glc*p*-(1→	2,3,6-Me_3_-Glc*p*	41.936	0.646	43, 87, 99, 101, 113, 117, 129, 131, 161, 173, 233
5	→3,4)-Glc*p*-(1→	2,6-Me_2_-Glc*p*	49.470	0.031	43, 87, 97, 117, 129, 149
6	→4,6)-Glc*p*-(1→	2,3-Me_2_-Glc*p*	54.456	0.070	43, 71, 85, 87, 99, 101, 117, 127, 159, 161, 201, 261

**Table 4:** Chemical shift assignments of the PMPS-A_1_.

Number	Glycosy Residues	Chemical Shifts (ppm)						
		H1/C1	H2/C2	H3/C3	H4/C4	H5/C5	H6a/C6	H6b
A	α-D-Glc*p*-1→	5.25	3.59	3.69	3.94	3.96	3.59	3.81
		100.92	71.53	73.62	70.2	71.27	61.89	
B	→3)-α- D-Glc*p*-(1→	5.18	3.48	3.52	3.42	3.61	3.66	3.73
		100.86	72.33	78.02	70.26	73.52	61.48	
C	→4-α-D-Glc*p*-(1→	5.3	3.51	3.89	3.57	3.77	3.75	3.7
		100.48	72.5	74.8	77.9	72.12	61.48	
D	→4,6)- α-D-Glc*p*-(1→	4.87	3.48	3.66	3.4	3.56	3.84	3.85
		99.48	72.45	73.61	76.69	70.28	69.04	
E	→3,4)-α-D-Glc*p*-(1→	5.26	3.49	3.74	3.73	3.84	3.65	3.77
		100.95	72.45	78	78.16	70.76	61.46	
F	→1)-β-D-Fru*f*-(2→	3.58, 3.69	nd	4.1	4.01	3.84	3.46	3.81
		61.27	105.62	77.74	76.59	70.76	64.81	

nd: not detected.

**Table 5:** Antibacterial activity of PMPS-A_1_.

Microorganisms	PMPS-A_1_ Concentration (mg/mL)				Penicillin (mg/mL)
	20	25	30	35	
*E. coli* 25,922	10 ± 0.45 ^b^	11 ± 0.98 ^b^	12 ± 1.03 ^b^	13 ± 0.89 ^b^	25.18 ± 1.24 ^a^
(MRSA) ATCC 29,213	11 ± 1.02 ^b^	12 ± 1.02 ^b^	13 ± 1.42 ^b^	14 ± 1.54 ^b^	26.17 ± 1.28 ^a^

The outside diameter of the cylinder is 7 mm (The unit is mm). Note: a and b suggest statistically significant (*p* < 0.05) differences in the size of inhibition zone of the same sample to different strains.

## Data Availability

The original contributions presented in the study are included in the article, further inquiries can be directed to the corresponding author.
